# Comparison of accuracy of two uncalibrated pulse contour cardiac output monitors in off-pump coronary artery bypass surgery patients using pulmonary artery catheter-thermodilution as a reference

**DOI:** 10.1186/s12871-021-01415-5

**Published:** 2021-07-10

**Authors:** Ramakrishna Mukkamala, Benjamin A. Kohl, Aman Mahajan

**Affiliations:** 1 Department of Anesthesiology and Perioperative Medicine and Department of Bioengineering, University of Pittsburgh, 408 Benedum Hall, 3700 O’Hara Street, Pittsburgh, PA 15261 USA; 2 Retia Medical, Valhalla, NY USA; 3 Department of Anesthesiology, Thomas Jefferson University, Philadelphia, PA USA; 4 Department of Anesthesiology and Perioperative Medicine and Department of Bioengineering, University of Pittsburgh, A-1305 Scaife Hall, 3550 Terrace Street, Pittsburgh, PA 15261 USA

**Keywords:** Argos, Cardiac output monitoring, FloTrac, Pulse contour analysis, Thermodilution

## Abstract

**Background:**

Cardiac output (CO) is a key measure of adequacy of organ and tissue perfusion, especially in critically ill or complex surgical patients. CO monitoring technology continues to evolve. Recently developed CO monitors rely on unique algorithms based on pulse contour analysis of an arterial blood pressure (ABP) waveform. The objective of this investigation was to compare the accuracy of two monitors using different methods of pulse contour analysis – the Retia Argos device and the Edwards Vigileo-FloTrac device – with pulmonary artery catheter (PAC)-thermodilution as a reference.

**Methods:**

Fifty-eight patients undergoing off-pump coronary artery bypass surgery formed the study cohort. A total of 572 triplets of CO measurements from each device – Argos, Vigileo-FloTrac (third generation), and thermodilution – were available before and after interventions (e.g., vasopressors, fluids, and inotropes). Bland–Altman analysis accounting for repeated measurements per subject and concordance analysis were applied to assess the accuracy of the CO values and intervention-induced CO changes of each pulse contour device against thermodilution. Cluster bootstrapping was employed to statistically compare the root-mean-squared-errors (RMSE = √(μ^2^ + σ^2^), where μ and σ are the Bland–Altman bias and precision errors) and concordance rates of the two devices.

**Results:**

The RMSE (mean (95% confidence intervals)) for CO values was 1.16 (1.00–1.32) L/min for the Argos device and 1.54 (1.33–1.77) L/min for the Vigileo-FloTrac device; the concordance rate for intervention-induced CO changes was 87 (82–92)% for the Argos device and 72 (65–78)% for the Vigileo-FloTrac device; and the RMSE for the CO changes was 17 (15–19)% for the Argos device and 21 (19–23)% for the Vigileo-FloTrac device (*p* < 0.0167 for all comparisons).

**Conclusions:**

In comparison with CO measured by the PAC, the Argos device proved to be more accurate than the Vigileo-FloTrac device in CO trending and absolute CO measurement in patients undergoing off-pump coronary artery bypass surgery.

**Supplementary Information:**

The online version contains supplementary material available at 10.1186/s12871-021-01415-5.

## Background

Cardiac output (CO) monitoring is routinely used to assist in the hemodynamic management of patients undergoing major surgery and in the intensive care unit. Devices that do not require calibration (with a reference CO method), or un-calibrated CO monitors, are commonly used in clinical practice. Of these, the pulse contour-based devices permit continuous CO monitoring via mathematical analysis of a peripheral arterial blood pressure (ABP) waveform [1]. Some of these devices detect pulse pressure as a marker of stroke volume and then multiply the pulse pressure with heart rate to compute the ratio of CO to the arterial compliance [2, 3]. Other devices fit an exponential to the ABP diastolic decay to determine the “Windkessel” time constant (τ = systemic vascular resistance times arterial compliance) and then divide mean ABP by the τ to compute the ratio of CO to the arterial compliance as well [4]. For example, the Vigileo-FloTrac device (Edwards Lifesciences, Irvine, USA) essentially determines pulse pressure times heart rate and then computes the arterial compliance term using various ABP waveform statistics and patient demographic information [2]. However, arterial wave reflection may cause the peripheral pulse pressure to change irrespective of stroke volume due to vasoconstriction or vasodilation and may also obscure exponential diastolic decays in peripheral ABP waveforms. While these devices have been extensively tested in human subjects, accuracy still remains a concern [1, 5–7]. Notably, none of the devices have emerged as being more accurate than another.

**Fig. 1:**
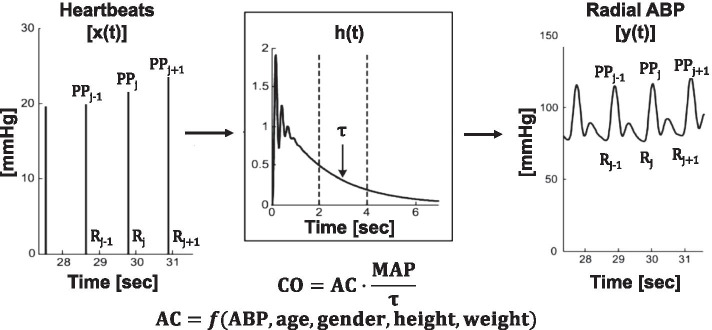
The Retia Argos device computes cardiac output (CO) by *multi-beat analysis* of a radial arterial blood pressure (ABP) waveform [8–13]. The ABP response to a single heartbeat [h(t)] is estimated from the ABP waveform segment over multiple beats via mathematical modeling. The Windkessel time constant τ, which equals the systemic vascular resistance times the arterial compliance (AC), is then determined by fitting an exponential to the tail end of this response once the faster wave reflection vanishes. Finally, CO is computed by dividing mean arterial pressure (MAP) by τ and determining the AC term from ABP and patient demographic information via a proprietary formula

The Argos device (Retia Medical, Valhalla, USA) is a new un-calibrated pulse contour device that has recently received US Food & Drug Administration (FDA) clearance [8]. The unique idea underlying this device is to apply a *multi-beat analysis (MBA™*) to model arterial wave reflection, as described previously [9–13] and shown in Fig. 1. Initially, the ABP response to a single heartbeat is estimated from a radial ABP waveform segment over multiple beats via mathematical modeling. The Windkessel time constant τ is then determined by fitting an exponential to the tail end of this response once the faster wave reflection vanishes. Finally, the arterial compliance term is determined from the ABP waveform and patient age, height, weight, and gender utilizing a proprietary formula.

Recently, Saugel and colleagues compared the Argos device with bolus thermodilution CO via a pulmonary artery catheter (PAC) in off-pump coronary artery bypass surgery patients [14]. The objective of the present study was to perform a secondary analysis of these data to compare the accuracy of the Argos device with the Vigileo-FloTrac device using thermodilution as a reference.

## Methods

### Existing patient data

All patient data for the current study have been previously described in detail [14] and were collected by Saugel and colleagues at University Medical Center Hamburg-Eppendorf as part of studies testing the accuracy of the Argos device. Their study was approved by the Ethics Committee of the Hamburg Medical Association (Germany), and all enrolled patients provided written, informed consent. The inclusion criteria were adults undergoing off-pump coronary artery bypass surgery who had clinical indications for radial and pulmonary artery catheterization. Exclusion criteria included the presence of severe arrhythmias or missing informed consent. Bolus thermodilution CO via a PAC (average of four consecutive injections of 10 ml ice-cold saline) was measured before and after up to six clinical interventions (e.g., fluids, vasopressors, and inotropes) in each patient, while the ABP waveform via a radial artery catheter was recorded at a sampling rate of 100 Hz throughout the surgery. The radial ABP waveform segments during the time periods of the reference thermodilution CO measurements were visually screened to exclude unreliable data [14]. A total of 572 pairs of ABP waveform segments and reference CO values from 58 patients (17% female, 70 ± 10 (mean ± SD) years in age, 1.74 ± 0.09 m in height, 81 ± 15 kg in weight, and 26.8 ± 4.2 kg/m^2^ in body mass index; see Table 1 in reference [14]) remained for analysis. The ABP waveform segments were entered off-line into the Argos device operating in 20-s mode, which outputs the average CO over the last 20-s every 5-s, by an independent contractor (see Acknowledgements section) [14]. The resulting CO values were then averaged over the time intervals of the reference CO values by the contractor. The contractor performed this work while blinded to the reference CO values.

All ABP waveforms were also entered off-line into the Vigileo-FloTrac device (software version 3.02) operating in 20-s mode by the same independent contractor. The data feeding procedure was similar to a procedure that was reviewed and approved by the US FDA (see Argos device Operator’s Manual [15]). The resulting CO values were then similarly averaged by the contractor to correspond to the reference CO values. The contractor was again blinded to the reference CO values.

### Secondary statistical data analysis

The total of 572 triplets of Argos, Vigileo-FloTrac, and thermodilution CO values as well as 509 corresponding triplets of intervention-induced changes in consecutive CO values (ΔCO = 100⋅(CO_after_—CO_before_)/CO_before_) from the 58 patients were provided after de-identification by Saugel and colleagues for secondary analysis. The provided data are included here – without alteration – as supplemental information (see Additional file 1). The local Institutional Review Board declared that this secondary analysis did not constitute human subjects research (Michigan State University Study ID: STUDY00002367).

Statistical analysis of the data was performed to compare the Argos and Vigileo-FloTrac devices in terms of the accuracy of their CO values (absolute measurement ability) and ΔCO values (trending ability) against the reference thermodilution CO values. The analysis was performed using the MATLAB software package (Natick, USA), and the code is also included here as supplemental information (see Additional file 2).

The accuracy of each of the Argos and Vigileo-FloTrac devices against reference thermodilution was individually assessed via Bland–Altman analysis of the CO and ΔCO values and standard concordance analysis of the ΔCO values (with a 15% exclusion zone). For the Bland–Altman analysis, repeated measurements per subject were taken into account via mixed effects modeling [16]. To quantify device errors, the root-mean-squared-errors (RMSEs) of the CO and ΔCO values of each device were computed as √(μ^2^ + σ^2^), where μ and σ are the Bland–Altman bias and precision errors, respectively.

The relative accuracies of the Argos and Vigileo-FloTrac devices were assessed via statistical comparisons of their CO RMSEs, ΔCO RMSEs, and concordance rates. Non-parametric cluster bootstrapping was utilized to calculate confidence intervals and make the comparisons [17]. Specifically, 10,000 random samples of patients with replacement of patients were taken from the collected data. The number of patients in each sample was 58, which is the number of patients in the clinical study, and all triplets of CO and ΔCO values from a patient were included in each sample to maintain the correlated data structure. For each sample, the CO RMSE, ΔCO RMSE, and concordance rate of each device were computed as described above, and the difference between each pair of the quantitative metrics of the two devices (Argos – Vigileo-FloTrac) was taken. Both 95% CIs of each quantitative metric of each device and X% CIs of each difference between the metrics of the two devices were then calculated from the corresponding distribution of 10,000 values via a standard percentile bootstrap. If the upper CI for the CO or ΔCO RMSE difference were less than 0 or the lower CI for the concordance rate difference were greater than 0, then the Argos device would be considered superior to the Vigileo-FloTrac device in terms of that particular metric. Since a total of three statistical comparisons were made, a Bonferroni correction was applied such that a two-sided p < 0.05/3 (i.e., X = 98.33) was considered statistically significant.

## Results

**Fig. 2:**
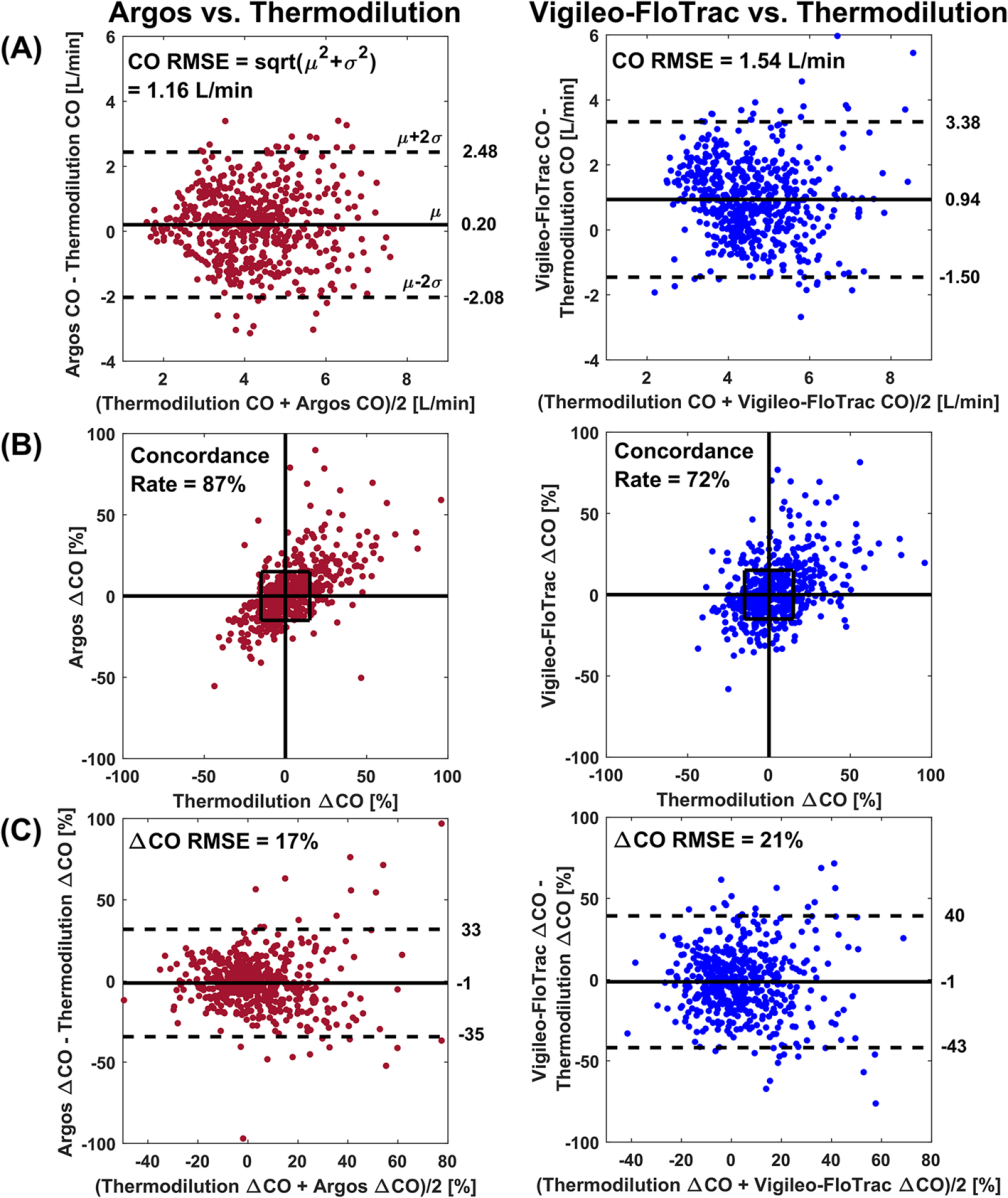
Absolute CO measurement and trending accuracy of the Retia Argos device and Edwards Vigileo-FloTrac device, each against reference pulmonary artery catheter (PAC)-thermodilution. **A** Bland–Altman plots of the CO values (*N* = 572 measurements from M = 58 patients). RMSE is root-mean-squared-error. **B** Concordance plots of intervention-induced CO changes (ΔCO = 100⋅(CO_after_—CO_before_)/CO_before_; *N* = 509 measurements from M = 58 patients). **C** Bland–Altman plots of the ΔCO values (*N* = 509 measurements from M = 58 patients.) The left plots in **A** and **B** match those in reference [14]

A total of 572 triplets of Argos, Vigileo-FloTrac, and thermodilution CO values as well as 509 corresponding triplets of intervention-induced changes in consecutive CO values (ΔCO = 100⋅(CO_after_—CO_before_)/CO_before_) from 58 patients were included in this study for secondary analysis. Figure 2A and C show Bland–Altman plots of the CO and ΔCO values of the Argos device versus reference thermodilution and of the Vigileo-FloTrac device versus the same reference, respectively. Figure 2B shows concordance plots of the ΔCO values of the Argos device versus reference thermodilution and of the Vigileo-FloTrac device versus this reference. Note that the Bland–Altman plot of the CO values and the concordance plot of the ΔCO values for the Argos device matched those reported by Saugel and colleagues (see Figs. 2 and 3 in reference [14]). The Table 1 shows statistical comparisons of the CO RMSE, ΔCO RMSE, and concordance rate of the Argos device versus the Vigileo-FloTrac device. This table also indicates the bias and precision error components of each RMSE.

**Table 1:** Comparison of absolute cardiac output (CO) and intervention-induced CO change (ΔCO) measurement accuracy of the Argos device and Vigileo-FloTrac device

Accuracy vs. Thermodilution	Argos	Vigileo-FloTrac
CO RMSE [L/min] μ ± 2σ	1.16 (1.00–1.32)* 0.20 ± 2.28	1.54 (1.33–1.77) 0.94 ± 2.44
Concordance Rate [%]	87 (82–92)*	72 (65–78)
ΔCO RMSE [%] μ ± 2σ	17 (15–19)* -1 ± 34	21 (19–23) -1 ± 42

RMSE = √(μ^2^ + σ^2^), where μ and σ are the Bland–Altman bias and precision errors, and stands for root-mean-squared-error. RMSE and concordance rate values are shown with 95% confidence intervals with the parentheses. The symbol * denotes *p* < 0.0167 versus Vigileo-FloTrac device. Confidence interval calculations and statistical tests were performed via cluster bootstrapping (*N* = 10,000 bootstrapped samples of the patient data). See Fig. 2 for raw data

The CO RMSE (mean (lower 95% CI-upper 95% CI)) was 1.16 (1.00–1.32) L/min for the Argos device and 1.54 (1.33–1.77) L/min for the Vigileo-FloTrac device (*p* < 0.0167). This difference was mainly due to the smaller bias error of the Argos device (see Fig. 2A and Table 1). The concordance rate was 87 (82–92)% for the Argos device and 72 (65–78)% for the Vigileo-FloTrac device (*p* < 0.0167). The ΔCO values of the Argos device also showed less scatter about the identity line in the concordance plots (see Fig. 2B). Consistent with these plots, the ΔCO RMSE was 17 (15–19)% for the Argos device and 21 (19–23)% for the Vigileo-FloTrac device (*p* < 0.0167). This difference was due to the smaller precision error (i.e., less scatter) of the Argos device (see Fig. 2C and Table 1).

## Discussion

Cardiac output monitoring allows improved hemodynamic management of critically ill and complex patients. The Retia Argos device is a new un-calibrated pulse contour device for CO monitoring that aims to improve accuracy by accounting for confounding arterial wave reflection in a radial ABP waveform with *multi-beat analysis* (see Fig. 1). In the present study, the Argos device was compared to the Edwards Vigileo-FloTrac device [18] using PAC-thermodilution CO as a reference via a secondary analysis of de-identified data from patients undergoing cardiac surgery. While the accuracy results of the Argos device against thermodilution (see left column of Fig. 2AB) were previously reported [14], the accuracy results of the Vigileo-FloTrac device against thermodilution are new (see right column of Fig. 2). These additional results importantly allowed for head-to-head comparisons of the two un-calibrated pulse contour devices on the exact same data (see entire Fig. 2 and Table 1). The aggregate results revealed that the Argos device was more accurate in absolute CO measurement and CO trending than the Vigileo-FloTrac device. Importantly, the testing of accuracy was performed using the standard clinical method (i.e., PAC-bolus thermodilution) as reference and before and after clinically relevant hemodynamic interventions (e.g., fluids, vasopressors, and inotropes).

The improvements in accuracy afforded by the Argos device could be seen visually in the Bland–Altman and concordance plots (see Fig. 2) and reached statistical significance (see Table 1). Most notably, the Argos device reduced the absolute CO RMSE by 25% and increased the concordance rate by 21% relative to the Vigileo-FloTrac device. To the best of our knowledge, few, if any, past studies have statistically shown that one un-calibrated pulse contour device is more accurate than another.

Standard Bland–Altman and concordance analyses were employed as a basis for the comparison of the two un-calibrated pulse contour devices [19]. Bland–Altman analysis of the L/min CO values of each device was performed to assess the absolute CO measurement accuracy. Bland–Altman analysis of the intervention-induced CO changes of each device was conducted to evaluate the trending accuracy in terms of both direction and magnitude of the CO change, while concordance analysis was performed to assess the directional trending accuracy alone. The repeated measurements per patient were accounted for by applying (i) mixed effects modeling [16] to compute the Bland–Altman bias and precision errors of each device and (ii) cluster bootstrapping [17] to compare RMSEs (i.e., a single number representing the aggregate of the two errors) and concordance rates of the two devices. Note that the popular percentage error (i.e., twice the precision error divided by the grand mean of the device and reference CO values) was not employed, as it is not a robust metric of accuracy. For example, as a device increasingly overestimates the reference measurements, the percentage error will progressively decrease – instead of increase. However, for completeness, the percentage errors of the Argos and Vigileo-FloTrac devices were both about 50% in this study. Also note that Bland–Altman analysis of the CO changes was preferred to polar plot analysis, which is more commonly used to assess the magnitude and direction of the CO change [19], because the error in the CO change yielded by the Bland–Altman analysis is more intuitive than the “angle” outputted by the polar plot analysis. In addition, percent CO changes rather than L/min CO changes were assessed, as L/min CO changes are confounded by the arterial compliance scale factor (see, e.g., Fig. 1) and are thus not a pure indicator of trending ability.

Acceptable levels of accuracy have previously been proposed including percentage errors within 30% or 40% and concordance rates exceeding 90% [6, 19]. The Argos device, but not the Vigileo-FloTrac device, achieved a concordance rate close to the proposed level and may thus offer value in CO trending. However, even though percentage error is not a robust accuracy metric, it may still be concluded that neither of the two pulse contour devices is interchangeable with PAC-thermodilution for absolute CO measurement.

A limitation of this study was that the third generation Vigileo-FloTrac device was used rather than the latest fourth generation device. However, the third generation device has often been used in clinical practice [18] and continues to be the subject of recent publications [20–24]. Further, while the fourth generation device appears to be improved in terms of measuring fast CO changes induced by phenylephrine boluses [25], it yielded an average percentage error of 47% and average concordance rate of 70% against PAC-thermodilution CO measurements in cardiac surgery patients in the OR [26, 27]. These results are similar to the results of the third generation device reported herein. Nevertheless, future comparisons of the Argos device and the fourth generation device should be conducted using the same patient data to draw definitive conclusions. Other limitations of the study were that the available patient cohort was confined to a relatively homogenous cardiac surgery population and that the accuracy difference between the two pulse contour devices could not be assessed for clinical significance. Additional studies are warranted to determine the relative accuracy and relative clinical value of these and other devices in hyperdynamic and other patient cohorts.

## Conclusions

In this study, the Retia Argos device with *multi-beat analysis* was more accurate than the Edwards Vigileo-FloTrac device in CO trending and absolute CO measurement in patients undergoing off-pump coronary artery bypass surgery. The accuracy attained in CO trending suggests the Argos device will potentially be more informative and useful if CO measurement is initiated when the arterial catheter is inserted rather than connecting the device to the catheter after a patient has already decompensated. A recent meta-analysis indicated that use of such un-calibrated pulse contour devices is associated with a decrease in postoperative morbidity [5]. Enhancing the CO measurement accuracy of the devices would not only provide a truer picture of the hemodynamic status of patients but could also potentially help to further improve patient outcomes.

## Supplementary Information

**Additional file 1.** Patient data provided by Saugel and colleagues. L/min cardiac output (CO) via reference pulmonary artery catheter-thermodilution (CO_TD (Lpm) sheet), Argos (CO_Retia (Lpm) sheet), and Vigileo-FloTrac (CO_Edwards (Lmin) sheet) devices. %CO change between consecutive L/min CO measurements via reference pulmonary artery catheter-thermodilution (d_TD (%) sheet), Argos (d_Retia (%) sheet), and Vigileo-FloTrac (d_Edwards (%) sheet) devices. Quality of blood pressure (BP) waveforms corresponding to L/min CO with accompanying legend (Quality sheet). Row corresponds to patient, and column denotes each measurement from the patient. PID – patient identification number; NaN – unavailable measurement in second through seventh sheets

**Additional file 2.** MATLAB code employed for secondary analysis of the patient data. Copy file content and paste into MATLAB as a script or at the prompt to reproduce data analysis results.

## Data Availability

All data and code employed in this study are provided through additional files as follows. • Patient data provided by Saugel and colleagues (Additional file 1). • MATLAB code employed for secondary analysis of the patient data (Additional file 2).

## Abbreviations

ABP: Arterial blood pressure
FDA: Food & Drug Administration
CI: Confidence intervals
CO: Cardiac output
ΔCO: Intervention-induced cardiac output change in percent
MAP: Mean arterial pressure
MBA: Multi-beat analysis
PAC: Pulmonary artery catheter
RMSE: Root-mean-squared-error (aggregate of the Bland–Altman bias error (μ) and precision error (σ))
τ: Windkessel time constant, which equals the systemic vascular resistance times the arterial compliance (AC)
