# Supplementary material for: Comparison of accuracy of two uncalibrated pulse contour cardiac output monitors in off-pump coronary artery bypass surgery patients using pulmonary artery catheter-thermodilution as a reference
Source: BMC Anesthesiol. 2021 Jul 10;21:189. doi: 10.1186/s12871-021-01415-5 (PMC8272317; doi:10.1186/s12871-021-01415-5)
Supplement: Supplementary file 2 — Additional file 2. MATLAB code employed for secondary analysis of the patient data. Copy file content and paste into MATLAB as a script or at the prompt to reproduce data analysis results. [file 12871_2021_1415_MOESM2_ESM.docx]

% This computer code is supplementary material to the paper: “Comparison of Accuracy of Two Uncalibrated Pulse Contour Cardiac Output Monitors in Off-pump Coronary Artery Bypass Surgery Patients using Pulmonary Artery Catheter-Thermodilution as a Reference” by Ramakrishna Mukkamala, Benjamin A. Kohl, and Aman Mahajan. To run this script, copy and paste this file into MATLAB (a script or at the prompt).

clear; clf;

% Importing the CO data from the excel file provided Saugel and colleagues

% Reading and converting the absolute CO values in L/min into a matrix

% (58 X 13). There are up to 12 values for each patient, which represent

% serial measurements before and after up to 6 clinical interventions.

cot = readtable('UKE_OR_final_data.xlsx','Sheet','CO_TD (Lpm)');

cot = cot{:,:};

cor = readtable('UKE_OR_final_data.xlsx','Sheet','CO_Retia (Lpm)');

cor = cor{:,:};

coe = readtable('UKE_OR_final_data.xlsx','Sheet','CO_Edwards (Lmin)');

coe = coe{:,:};

% Reading and converting the percent CO changes (= 100(CO_after -

% CO_before)/CO_before) into a matrix (58 X 12). There are up to 11

% values for each patient, which represent changes in consecutive absolute

% CO values.

dcot = readtable('UKE_OR_final_data.xlsx','Sheet','d_TD (%)');

dcot = dcot{:,:};

dcor = readtable('UKE_OR_final_data.xlsx','Sheet','d_Retia (%)');

dcor = dcor{:,:};

dcoe = readtable('UKE_OR_final_data.xlsx','Sheet','d_Edwards (%)');

dcoe = dcoe{:,:};

% Evaluating the absolute CO measurement accuracy and the CO trending

% accuracy of the Argos device against pulmonary artery catheter-

% thermodilution and of the Vigileo-FloTrac device against thermodilution.

% Converting the absolute CO value matrix into another matrix (696 X 4)

% where the columns represent the patient number, thermodilution values,

% Argos values, and Vigileo-FloTrac values.

coo = [];

for p=1:length(cot)

coo = [coo; p*ones(12,1) cot(p,2:end)' cor(p,2:end)' coe(p,2:end)'];

end

% Converting the percent CO change matrix into another matrix (638 X 4)

% where the columns represent the patient number, thermodilution changes,

% Argos changes, and Vigileo-FloTrac changes.

dcoo = [];

for k=1:length(dcot)

dcoo = [dcoo; k*ones(11,1) dcot(k,2:end)' dcor(k,2:end)' dcoe(k,2:end)'];

end

% Discarding the NaNs (which represent unavailable data or unreliable data

% as determined by Saugel and colleagues) in the absolute CO value matrix

% to arrive at a reduced matrix (572 X 4) comprising the final data for

% analysis.

co = [];

for s = 1:length(coo)

if (max(isnan(coo(s,:)))<1)

co = [co; coo(s,:)];

end

end

% Discardiing the NaNs in the percent CO change matrix to arrive at a

% reduced matrix (509 X 4) for analysis.

dco = [];

for l = 1:length(dcoo)

if (max(isnan(dcoo(l,:)))<1)

dco = [dco; dcoo(l,:)];

end

end

% Computing the BA bias and precision errors and RMSE accounting for

% repeated measures as described by Bland and Altman in [14] for the

% absolute CO values of the Argos device.

tcore = table(co(:,3)-co(:,2),co(:,1),'VariableNames',{'errorr','patient'});

re = fitlme(tcore,'errorr ~ 1 + (1|patient)','FitMethod','REML');

[psir,mser] = covarianceParameters(re);

display(['Bias error, precision error, and RMSE of CO values in L/min for Argos device:']);

mur = fixedEffects(re)

stdr = sqrt(cell2mat(psir)+mser)

rmser = sqrt(mur^2+cell2mat(psir)+mser)

% Computing the BA bias and precision errors and RMSE accounting for

% repeated measures for the absolute CO values of the Vigileo-FloTrac

% device.

tcoee = table(co(:,4)-co(:,2),co(:,1),'VariableNames',{'errore','patient'});

ee = fitlme(tcoee,'errore ~ 1 + (1|patient)','FitMethod','REML');

[psie,msee] = covarianceParameters(ee);

display(['Bias error, precision error, and RMSE of CO values in L/min for Vigileo-FloTrac device:']);

mue = fixedEffects(ee)

stde = sqrt(cell2mat(psie)+msee)

rmsee = sqrt(mue^2+cell2mat(psie)+msee)

% Computing the concordance rate with 15% exclusion zone for the percent CO

% changes of the Argos device.

countn = 0;

countd = 0;

for m = 1:length(dco)

if ((abs(dco(m,2))>15 | abs(dco(m,3))>15))

countd = countd+1;

if (sign(dco(m,2)*dco(m,3))==1)

countn=countn+1;

end

end

end

display(['Concordance rate of percent CO changes for Argos device:']);

concordancer = 100*countn/countd

% Computing the concordance rate with 15% exclusion zone for the percent CO

% changes of the Vigileo-FloTrac device.

countnn = 0;

countdd = 0;

for n = 1:length(dco)

if ((abs(dco(n,2))>15 | abs(dco(n,4))>15))

countdd = countdd+1;

if (sign(dco(n,2)*dco(n,4))==1)

countnn=countnn+1;

end

end

end

display(['Concordance rate of percent CO changes for Vigile-FloTrac device:']);

concordancee = 100*countnn/countdd

% Computing the BA bias and precision errors and RMSE accounting for

% repeated measures for the percent CO changes of the Argos device.

tdcore = table(dco(:,3)-dco(:,2),dco(:,1),'VariableNames',{'derrorr','patient'});

dre = fitlme(tdcore,'derrorr ~ 1 + (1|patient)','FitMethod','REML');

[psidr,msedr] = covarianceParameters(dre);

display(['Bias error, precision error, and RMSE of percent CO changes for Argos device:']);

mudr = fixedEffects(dre)

stddr = sqrt(cell2mat(psidr)+msedr)

rmsedr = sqrt(mudr^2+cell2mat(psidr)+msedr)

% Computing the BA bias and precision errors and RMSE accounting for

% repeated measures for the percent CO changes of the Vigileo-FloTrac

% device.

tdcoee = table(dco(:,4)-dco(:,2),dco(:,1),'VariableNames',{'derrore','patient'});

dee = fitlme(tdcoee,'derrore ~ 1 + (1|patient)','FitMethod','REML');

[pside,msede] = covarianceParameters(dee);

display(['Bias error, precision error, and RMSE of percent CO changes for Vigileo-FloTrac device:']);

mude = fixedEffects(dee)

stdde = sqrt(cell2mat(pside)+msede)

rmsede = sqrt(mude^2+cell2mat(pside)+msede)

% Making the BA plots and concordance plots for the Argos and

% Vigileo-FloTrac devices using the quantitative metrics calculated above.

% Making BA plots for the absolute CO values of each device against

% thermodilution.

figure(1);

subplot(321);

plot((co(:,2)+co(:,3))/2,co(:,3)-co(:,2),'o');

axis([1 9 -4 6]);

hold;

plot([0 10],[mur mur],'k');

plot([0 10],[mur+1.96*stdr mur+1.96*stdr],'k--');

plot([0 10],[mur-1.96*stdr mur-1.96*stdr],'k--');

title('Argos vs. Thermodilution');

ylabel('Argos CO - Thermodilution CO [L/min]');

xlabel('(Thermodilution CO + Argos CO)/2 [L/min]');

subplot(322);

plot((co(:,2)+co(:,4))/2,co(:,4)-co(:,2),'o');

hold;

plot([0 10],[mue mue],'k');

plot([0 10],[mue+1.96*stde mue+1.96*stde],'k--');

plot([0 10],[mue-1.96*stde mue-1.96*stde],'k--');

axis([1 9 -4 6]);

title('Vigileo-FloTrac vs. Thermodilution');

ylabel('Vigileo-FloTrac CO - Thermodilution CO [L/min]');

xlabel('(Thermodilution CO + Vigileo-FloTrac CO)/2 [L/min]');

% Making concordance plots for the percent CO changes of each device

% against thermodilution.

subplot(323);

plot(dco(:,2),dco(:,3),'o');

axis([-100 100 -100 100]);

hold;

plot([0 0],[-110 110],'k');

plot([-110 110],[0 0],'k');

plot([-15 -15],[-15 15],'k');

plot([15 15],[-15 15],'k');

plot([-15 15],[15 15],'k');

plot([-15 15],[-15 -15],'k');

ylabel('Argos \DeltaCO [%]');

xlabel('Thermodilution \DeltaCO [%]');

subplot(324);

plot(dco(:,2),dco(:,4),'o');

axis([-100 100 -100 100]);

hold;

plot([0 0],[-110 110],'k');

plot([-110 110],[0 0],'k');

plot([-15 -15],[-15 15],'k');

plot([15 15],[-15 15],'k');

plot([-15 15],[15 15],'k');

plot([-15 15],[-15 -15],'k');

ylabel('Vigileo-FloTrac \DeltaCO [%]');

xlabel('Thermodilution \DeltaCO [%]');

% Making BA plots for the percent CO changes of each device against

% thermodilution.

subplot(325);

plot((dco(:,2)+dco(:,3))/2,dco(:,3)-dco(:,2),'o');

hold;

plot([-50 100],[mudr mudr],'k');

plot([-50 100],[mudr+1.96*stddr mudr+1.96*stddr],'k--');

plot([-50 100],[mudr-1.96*stddr mudr-1.96*stddr],'k--');

axis([-50 80 -100 100]);

ylabel('Argos \DeltaCO - Thermodilution \DeltaCO [%]');

xlabel('(Thermodilution \DeltaCO + Argos \DeltaCO)/2 [%]');

subplot(326);

plot((dco(:,2)+dco(:,4))/2,dco(:,4)-dco(:,2),'o');

axis([-50 80 -100 100]);

hold;

plot([-50 100],[mude mude],'k');

plot([-50 100],[mude+1.96*stdde mude+1.96*stdde],'k--');

plot([-50 100],[mude-1.96*stdde mude-1.96*stdde],'k--');

ylabel('Vigileo-FloTrac \DeltaCO - Thermodilution \DeltaCO [%]');

xlabel('(Thermodilution \DeltaCO + Vigileo-FloTrac \DeltaCO)/2 [%]');

% Comparing the absolute CO measurement accuracy and the CO trending

% accuracy of the Argos device with the Vigileo-FloTrac device.

% Performing cluster bootstrapping [15] via 10,000 bootstrapped samples

for i = 1:10000

i

% Generating the ith bootstrap sample by randomly drawing 58 patients

% with replacement from the original clinical data matrices.

cotbs = [];

corbs = [];

coebs = [];

dcotbs = [];

dcorbs = [];

dcoebs = [];

for j = 1:58

sub = round(0.5 + 58*rand(1,1));

cotbs = [cotbs; cot(sub,:)];

corbs = [corbs; cor(sub,:)];

coebs = [coebs; coe(sub,:)];

dcotbs = [dcotbs; dcot(sub,:)];

dcorbs = [dcorbs; dcor(sub,:)];

dcoebs = [dcoebs; dcoe(sub,:)];

end

% Converting the ith bootstrapped absolute CO value matrix into another

% matrix where the columns represent the patient number, thermodilution

% values, Argos values, and Vigileo-FloTrac values.

cobso = [];

for p=1:length(cot)

cobso = [cobso; p*ones(12,1) cotbs(p,2:end)' corbs(p,2:end)' coebs(p,2:end)'];

end

% Converting the ith bootstrapped percent CO change matrix into another

% matrix where the columns represent the patient number, thermodilution

% changes, Argos changes, and Vigileo-FloTrac changes.

dcobso = [];

for k=1:length(dcot)

dcobso = [dcobso; k*ones(11,1) dcotbs(k,2:end)' dcorbs(k,2:end)' dcoebs(k,2:end)'];

end

% Discardiing the NaNs in the ith bootstrapped absolute CO value matrix

% to arrive at a reduced matrix of final data for analysis.

cobs = [];

for s = 1:length(cobso)

if (max(isnan(cobso(s,:)))<1)

cobs = [cobs; cobso(s,:)];

end

end

% Discardiing the NaNs in the ith bootstrapped percent CO change matrix

% to arrive at a reduced matrix for analysis.

dcobs = [];

for l = 1:length(dcobso)

if (max(isnan(dcobso(l,:)))<1)

dcobs = [dcobs; dcobso(l,:)];

end

end

% Computing the BA bias and precision errors and RMSE accounting for

% repeated measures for the ith bootstrapped sample of absolute CO

% values of the Argos device.

tcorebs = table(cobs(:,3)-cobs(:,2),cobs(:,1),'VariableNames',{'errorr','patient'});

rebs = fitlme(tcorebs,'errorr ~ 1 + (1|patient)','FitMethod','REML');

[psi,mse] = covarianceParameters(rebs);

mu = fixedEffects(rebs);

rmser(i) = sqrt(mu^2+cell2mat(psi)+mse);

% Computing the BA bias and precision errors and RMSE accounting for

% repeated measures for the ith bootstrapped sample of absolute CO

% values of the Vigileo-FloTrac device.

tcoeebs = table(cobs(:,4)-cobs(:,2),cobs(:,1),'VariableNames',{'errore','patient'});

eebs = fitlme(tcoeebs,'errore ~ 1 + (1|patient)','FitMethod','REML');

[psi,mse] = covarianceParameters(eebs);

mu = fixedEffects(eebs);

rmsee(i) = sqrt(mu^2+cell2mat(psi)+mse);

% Computing the concordance rate with 15% exclusion zone for the ith

% bootstrapped sample of percent CO changes of the Argos device.

countn = 0;

countd = 0;

for m = 1:length(dcobs)

if ((abs(dcobs(m,2))>15 | abs(dcobs(m,3))>15))

countd = countd+1;

if (sign(dcobs(m,2)*dcobs(m,3))==1)

countn=countn+1;

end

end

end

concordancer(i) = 100*countn/countd;

% Computing the concordance rate with 15% exclusion zone for the ith

% bootstrapped sample of percent CO changes of the Vigileo-FloTrac

% device.

countnn = 0;

countdd = 0;

for n = 1:length(dcobs)

if ((abs(dcobs(n,2))>15 | abs(dcobs(n,4))>15))

countdd = countdd+1;

if (sign(dcobs(n,2)*dcobs(n,4))==1)

countnn=countnn+1;

end

end

end

concordancee(i) = 100*countnn/countdd;

% Computing the BA bias and precision errors and RMSE accounting for

% repeated measures for the ith bootstrapped sample of percent CO

% changes of the Argos device.

tdcorebs = table(dcobs(:,3)-dcobs(:,2),dcobs(:,1),'VariableNames',{'derrorr','patient'});

drebs = fitlme(tdcorebs,'derrorr ~ 1 + (1|patient)','FitMethod','REML');

[psi,mse] = covarianceParameters(drebs);

mu = fixedEffects(drebs);

rmsedr(i) = sqrt(mu^2+cell2mat(psi)+mse);

% Computing the BA bias and precision errors and RMSE accounting for

% repeated measures for the ith bootstrapped sample of percent CO

% changes of the Vigileo-Flotrac device.

tdcoeebs = table(dcobs(:,4)-dcobs(:,2),dcobs(:,1),'VariableNames',{'derrore','patient'});

deebs = fitlme(tdcoeebs,'derrore ~ 1 + (1|patient)','FitMethod','REML');

[psi,mse] = covarianceParameters(deebs);

mu = fixedEffects(deebs);

rmsede(i) = sqrt(mu^2+cell2mat(psi)+mse);

end

% Computing the 98.33% CIs for the Argos CO RMSE - Vigile-FloTrac CO RMSE

% via a standard percentile bootstrap.

drmse = sort(rmser-rmsee);

display(['Lower and upper 98.33% CIs for Argos CO RMSE - Vigileo-FloTrac CO RMSE:']);

drmse(83)

drmse(9917)

% Computing the p-value for the statistical comparison of the Argos CO RMSE

% vs. the Vigileo-FloTrac CO RMSE using a formula given by Bland and

% Altman [BMJ 2011;343:d2304].

Z = abs(mean(drmse)/((drmse(9750)-drmse(250))/(2*1.96)));

display(['p value for comparison of Argos CO RMSE vs. Vigile-FloTrac CO RMSE:']);

pr = exp(-0.717*Z-0.416*Z^2)

% Computing the 98.33% CIs for the Argos concordance rate - Vigile-FloTrac

% concordance rate.

dconcordance = sort(concordancer-concordancee);

display(['Lower and upper 98.33% CIs for Argos concordance rate - Vigileo-FloTrac concordance rate:']);

dconcordance(83)

dconcordance(9917)

% Computing the p-value for the statistical comparison of the Argos

% concordance rate vs. the Vigileo-FloTrac concordance rate.

Z = abs(mean(dconcordance)/((dconcordance(9750)-dconcordance(250))/(2*1.96)));

display(['p value for comparison of Argos concordance rate vs. Vigile-FloTrac concordance rate:']);

pc = exp(-0.717*Z-0.416*Z^2)

% Computing the 98.33% CIs for the Argos percent CO change RMSE -

% Vigile-FloTrac percent CO change RMSE.

drmsed = sort(rmsedr-rmsede);

display(['Lower and upper 98.33% CIs for Argos percent CO change RMSE - Vigileo-FloTrac percent CO change RMSE:']);

drmsed(83)

drmsed(9917)

% Computing the p-value for the statistical comparison of the Argos

% percent CO change RMSE vs. the Vigileo-FloTrac percent CO change RMSE.

Z = abs(mean(drmsed)/((drmsed(9750)-drmsed(250))/(2*1.96)));

display(['p value for comparison of Argos percent CO change RMSE vs. Vigile-FloTrac percent CO change RMSE:']);

pdr = exp(-0.717*Z-0.416*Z^2)

% Making bar graphs to illustrate the comparison of each of the three

% quantitative metrics of the two devices.

figure(2);

% Making the bar graph for the CO RMSEs.

subplot(311);

% Computing the mean and 95% CIs for the CO RMSEs of the Argos device and

% of the Vigileo-FloTrac device.

srmser = sort(rmser);

srmsee = sort(rmsee);

mure = mean(srmser);

muee = mean(srmsee);

sigmarel = srmser(250);

sigmareh = srmser(9750);

sigmaeel = srmsee(250);

sigmaeeh = srmsee(9750);

X = categorical({'Argos','Vigileo-FloTrac'});

X = reordercats(X,{'Argos','Vigileo-FloTrac'});

bar(X(1),mure);

hold on;

bar(X(2),muee);

er = errorbar(X,[mure muee],[sigmarel-mure sigmaeel-muee],[sigmareh-mure sigmaeeh-muee]);

er.Color = [0 0 0];

er.LineStyle = 'none';

ylabel('CO RMSE [L/min]')

title('Argos vs. Vigileo-FloTrac with Thermodilution as Reference')

ylim([0 2]);

% Making te bar graph for the concordance rates.

subplot(312);

% Computing the mean and 95% CIs for the concordance rates of the Argos

% device and of the Vigileo-FloTrac device.

sconcordancer = sort(concordancer);

sconcordancee = sort(concordancee);

murc = mean(sconcordancer);

muec = mean(sconcordancee);

sigmarcl = sconcordancer(250);

sigmarch = sconcordancer(9750);

sigmaecl = sconcordancee(250);

sigmaech = sconcordancee(9750);

X = categorical({'Argos','Vigileo-FloTrac'});

X = reordercats(X,{'Argos','Vigileo-FloTrac'});

bar(X(1),murc);

hold on;

bar(X(2),muec);

er = errorbar(X,[murc muec],[sigmarcl-murc sigmaecl-muec],[sigmarch-murc sigmaech-muec]);

er.Color = [0 0 0];

er.LineStyle = 'none';

ylabel('Concordance Rate [%]')

ylim([50 100]);

% Making the bar graph for the percent CO change RMSEs.

subplot(313);

% Computing the mean and 95% cIs for the percent CO change RMSEs of the

% Argos device and of the Vigileo-FloTrac device.

srmsedr = sort(rmsedr);

srmsede = sort(rmsede);

mudre = mean(srmsedr);

mudee = mean(srmsede);

sigmadrel = srmsedr(250);

sigmadreh = srmsedr(9750);

sigmadeel = srmsede(250);

sigmadeeh = srmsede(9750);

X = categorical({'Argos','Vigileo-FloTrac'});

X = reordercats(X,{'Argos','Vigileo-FloTrac'});

bar(X(1),mudre);

hold on;

bar(X(2),mudee);

er = errorbar(X,[mudre mudee],[sigmadrel-mudre sigmadeel-mudee],[sigmadreh-mudre sigmadeeh-mudee]);

er.Color = [0 0 0];

er.LineStyle = 'none';

ylabel('\DeltaCO RMSE [%]');

ylim([0 25]);
